# An *O*(*m* log *n*) algorithm for branching bisimilarity on labelled transition systems

**DOI:** 10.1007/978-3-030-45237-7_1

**Published:** 2020-03-13

**Authors:** David N. Jansen, Jan Friso Groote, Jeroen J. A. Keiren, Anton Wijs

**Affiliations:** 8 Johannes Kepler University, Linz, Austria; 9 University of Birmingham, Birmingham, UK; 10 State Key Laboratory of Computer Science, Institute of Software, Chinese Academy of Sciences, Beijing, China; 11 Department of Mathematics and Computer Science, Eindhoven University of Technology, Eindhoven, The Netherlands

**Keywords:** Branching bisimilarity, Algorithm, Labelled transition systems

## Abstract

Branching bisimilarity is a behavioural equivalence relation on labelled transition systems (LTSs) that takes internal actions into account. It has the traditional advantage that algorithms for branching bisimilarity are more efficient than ones for other weak behavioural equivalences, especially weak bisimilarity. With *m* the number of transitions and *n* the number of states, the classic ${O\left( {m n}\right) }$ algorithm was recently replaced by an $O({m (\log \left| { Act }\right| + \log n)})$ algorithm [9], which is unfortunately rather complex. This paper combines its ideas with the ideas from Valmari [20], resulting in a simpler $O({m \log n})$ algorithm. Benchmarks show that in practice this algorithm is also faster and often far more memory efficient than its predecessors, making it the best option for branching bisimulation minimisation and preprocessing for calculating other weak equivalences on LTSs.

### 1 Introduction

Branching bisimilarity [8] is an alternative to weak bisimilarity [17]. Both equivalences allow the reduction of labelled transition systems (LTSs) containing transitions labelled with internal actions, also known as silent, hidden or $\tau$-actions.

One of the distinct advantages of branching bisimilarity is that, from the outset, an efficient algorithm has been available [10], which can be used to calculate whether two states are equivalent and to calculate a quotient LTS. It has complexity $O(mn)$ with $m$ the number of transitions and $n$ the number of states. It is more efficient than classic algorithms for weak bisimilarity, which use transitive closure (for instance, [16] runs in $O\big(n^2 m \log n + mn^{2.376}\big)$, where $n^{2.376}$ is the time for computing the transitive closure), and algorithms for weak simulation equivalence (strong simulation equivalence can be computed in $O(mn)$ [12], and for weak simulation equivalence first the transitive closure needs to be computed). The algorithm is also far more efficient than algorithms for trace-based equivalence notions, such as (weak) trace equivalence or weak failure equivalence [16].

Branching bisimilarity also enjoys the nice mathematical property that there exists a canonical quotient with a minimal number of states and transitions (contrary to, for instance, trace-based equivalences). Additionally, as branching bisimilarity is coarser than virtually any other behavioural equivalence taking internal actions into account [7], it is ideal for preprocessing. In order to calculate a desired equivalence, one can first reduce the behaviour modulo branching bisimilarity, before applying a dedicated algorithm on the often substantially reduced transition system. In the mCRL2 toolset [5] this is common practice.

In [9,11] an algorithm to calculate stuttering equivalence on Kripke structures with complexity $O(m \log n)$ was proposed. Stuttering equivalence essentially differs from branching bisimilarity in the fact that transitions do not have labels and as such all transitions can be viewed as internal. In these papers it was shown that branching bisimilarity can be calculated by translating LTSs to Kripke structures, encoding the labels of transitions into labelled states following [6,19]. This led to an $O(m(\log |Act| + \log n))$ or $O(m \log m)$ algorithm for branching bisimilarity.

Besides the time complexity, the algorithm in [9,11] has two disadvantages. First, the translation to Kripke structures introduces a new state and a new transition per action label and target state of a transition, which increases the memory required to calculate branching bisimilarity. This made it far less memory efficient than the classical algorithm of [10], and this was perceived as a substantial practical hindrance. For instance, when reducing systems consisting of tens of millions of states, such as [2], memory consumption is the bottleneck. Second, the algorithm in [9,11] is very complex. To illustrate the complexity, implementing it took approximately half a person-year.

**Contributions.** We present an algorithm for branching bisimilarity that runs directly on LTSs in $O(m \log n)$ time and that is simpler than the algorithm of [9,11]. To achieve this we use an idea from Valmari and Lehtinen [20,21] for strong bisimilarity. The standard Paige–Tarjan algorithm [18], which has $O(m \log n)$ time complexity for strong bisimilarity on Kripke structures, registers work done in a separate partition of states. Valmari [20] observed that this leads to complexity $O(m \log m)$ on LTSs and proposed to use a partition of transitions, whose elements he (and we) calls *bunches,* to register work done. This reduces the time complexity on LTSs to $O(m \log n)$.

Using this idea we design our more straightforward algorithm for branching bisimilarity on LTSs. Essentially, this makes the maintenance of action labels particularly straightforward and allows to simplify the handling of new, so-called, bottom states [10]. It also leads to a novel main invariant, which we formulate as Invariant 1. It allows us to prove the correctness of the algorithm in a far more straightforward way than before.

We have proven the correctness and complexity of the algorithm in detail [14] and demonstrate that it outperforms all preceding algorithms both in time and space when the LTSs are sizeable. This is illustrated with more than 30 example LTSs. This shows that the new algorithm pushes the state-of-the-art in comparing and minimising the behaviour of LTSs w.r.t. weak equivalences, either directly (branching bisimilarity) or using the form of a preprocessing step (for other weak equivalences).

Despite the fact that this new algorithm is more straightforward than the previous $O(m(\log |Act| + \log n))$ algorithm [9], the implementation of the algorithm is still not easy. To guard against implementation errors, we extensively applied random testing, comparing the output with that of other algorithms. The algorithms and their source code are freely available in the mCRL2 toolset [5].

**Overview of the article.** In Section 2 we provide the definition of LTSs and branching bisimilarity. In Section 3 we provide the core algorithm with high-level data structures, correctness and complexity. The subsequent section presents the procedure for splitting blocks, which can be presented as an independent pair of coroutines. Section 5 presents some benchmarks. Proofs and implementation details are omitted in this paper, and can be found in [14].

### 2 Branching bisimilarity

In this section we define labelled transition systems and branching bisimilarity.

**Definition 1 (Labelled transition system).** *A* labelled transition system *(LTS) is a triple $A = (S, Act, \rightarrow)$ where*

1. *$S$ is a finite set of* states. *The number of states is denoted by $n$.*
2. *$Act$ is a finite set of actions including the* internal action *$\tau$.*
3. *$\rightarrow \subseteq S \times Act \times S$ is a* transition relation. *The number of transitions is necessarily finite and denoted by $m$.*

It is common to write $t \xrightarrow{a} t'$ for $(t, a, t') \in \rightarrow$. With slight abuse of notation we write $t \xrightarrow{a} t' \in T$ instead of $(t, a, t') \in T$ for $T \subseteq \rightarrow$. We also write $t \xrightarrow{a} Z$ for the set of transitions $\{t \xrightarrow{a} t' \mid t' \in Z\}$, and $Z \xrightarrow{a} Z'$ for the set $\{t \xrightarrow{a} t' \mid t \in Z, t' \in Z'\}$. We call all actions except $\tau$ the *visible* actions. If $t \xrightarrow{a} t'$, we say that from $t$, the state $t'$, the action $a$, and the transition $t \xrightarrow{a} t'$ are *reachable.*

**Definition 2 (Branching bisimilarity).** *Let $A = (S, Act, \rightarrow)$ be an LTS. We call a relation $R \subseteq S \times S$ a* branching bisimulation relation *iff it is symmetric and for all $s, t \in S$ such that $s \mathrel{R} t$ and all transitions $s \xrightarrow{a} s'$ we have:*

1. *$a = \tau$ and $s' \mathrel{R} t$, or*
2. *there is a sequence $t \xrightarrow{\tau} \cdots \xrightarrow{\tau} t' \xrightarrow{a} t''$ such that $s \mathrel{R} t'$ and $s' \mathrel{R} t''$.*

*Two states $s$ and $t$ are* branching bisimilar, *denoted by $s \leftrightarrow_b t$, iff there is a branching bisimulation relation $R$ such that $s \mathrel{R} t$.*

Note that branching bisimilarity is an equivalence relation. Given an equivalence relation $R$, a transition $s \xrightarrow{a} t$ is called *inert* iff $a = \tau$ and $s \mathrel{R} t$. If $t \xrightarrow{\tau} t_1 \xrightarrow{\tau} \cdots \xrightarrow{\tau} t_{n-1} \xrightarrow{\tau} t_n \xrightarrow{a} t'$ such that $t \mathrel{R} t_i$ for $1 \leq i \leq n$, we say that the state $t_n$, the action $a$, and the transition $t_n \xrightarrow{a} t'$ are *inertly reachable* from $t$.

The equivalence classes of branching bisimilarity partition the set of states.

**Definition 3 (Partition).** *For a set $X$ a* partition *$\Pi$ of $X$ is a disjoint cover of $X$, i.e., $\Pi = \{B_i \subseteq X \mid B_i \neq \emptyset, 1 \leq i \leq k\}$ such that $B_i \cap B_j = \emptyset$ for all $1 \leq i < j \leq k$ and $X = \bigcup_{1 \leq i \leq k} B_i$.*

*A partition $\Pi'$ is a* refinement *of $\Pi$ iff for every $B' \in \Pi'$ there is some $B \in \Pi$ such that $B' \subseteq B$.*

We will often use that a partition $\Pi$ induces an equivalence relation in the following way: $s \equiv_\Pi t$ iff there is some $B \in \Pi$ containing both $s$ and $t$.

## 3 The algorithm

In this section we present the core algorithm. In the next section we deal with the actual splitting of blocks in the partition. We start off with an abstract description of this core part.

### 3.1 High-level description of the algorithm

The algorithm is a partition refinement algorithm. It iteratively refines two partitions $\Pi_s$ and $\Pi_t$. Partition $\Pi_s$ is a partition of states in $S$ that is coarser than branching bisimilarity. We refer to the elements of $\Pi_s$ as *blocks,* typically denoted using $B$. Partition $\Pi_t$ partitions the non-inert transitions of $\rightarrow$, where inertness is interpreted with respect to $\equiv_{\Pi_s}$. We refer to the elements of $\Pi_t$ as *bunches,* typically denoted using $T$.

The partition of transitions $\Pi_t$ records the current knowledge about transitions. Transitions are in different bunches iff the algorithm has established that they cannot simulate each other (i.e., they cannot serve as $s \xrightarrow{a} s'$ and $t' \xrightarrow{a} t''$ in Definition 2).

The partition of states $\Pi_s$ records the current knowledge about branching bisimilarity. Two states are in different blocks iff the algorithm has found a proof that they are not branching bisimilar (this is formalised in Invariant 3). This implies that $\Pi_s$ must be such that states with outgoing transitions in different combinations of bunches are in different blocks (Invariant 1).

Before performing partition refinement, the LTS is preprocessed to contract $\tau$-strongly connected components (SCCs) into a single state without a $\tau$-loop. This step is valid as all states in a $\tau$-SCC are branching bisimilar. Consequently, every block has *bottom states,* i.e., states without outgoing inert $\tau$-transitions [10].

The core invariant of the algorithm says that if one state in a block can inertly reach a transition in a bunch, all states in that block can inertly reach a transition in this bunch. This can be formulated in terms of bottom states:

**Invariant 1 (Bunches).** *$\Pi_s$ is stable under $\Pi_t$, i.e., if a bunch $T \in \Pi_t$ contains a transition with its source state in a block $B \in \Pi_s$, then every bottom state in block $B$ has a transition in bunch $T$.*

The initial partitions $\Pi_s$ and $\Pi_t$ are the coarsest partitions that satisfy Invariant 1. $\Pi_t$ starts with a single bunch consisting of all non-inert transitions. Then, in $\Pi_s$ we need to separate states with some transition in this bunch from those without. We define $B_{\text{vis}}$ to be the set of states from which a visible transition is inertly reachable, and $B_{\text{invis}}$ to be the other states. Then $\Pi_s = \{B_{\text{vis}}, B_{\text{invis}}\} \setminus \{\emptyset\}$.

Transitions in a bunch may have different labels or go to different blocks. In that case, the bunch can be split as these transitions cannot simulate each other. If we manage to achieve the situation where all transitions in a bunch have the same label and go to the same target block, the obtained partition turns out to be a branching bisimulation. Therefore, we want to split each bunch into so-called action-block-slices defined below. We also immediately define some other sets derived from $\Pi_t$ and $\Pi_s$ as we require them in our further exposition. So, we have:

- The *action-block-slices,* i.e., the transitions in $T$ with label $a$ ending in $B'$:
  $T_{\xrightarrow{a} B'} = \{s \xrightarrow{a} s' \in T \mid s' \in B'\}$.
- The *block-bunch-slices,* i.e., the transitions in $T$ starting in $B$:
  $T_{B\rightarrow} = \{s \xrightarrow{b} s' \in T \mid s \in B\}$.
- A block-bunch-slice intersected with an action-block-slice:
  $T_{B \xrightarrow{a} B'} = T_{B\rightarrow} \cap T_{\xrightarrow{a} B'} = \{s \xrightarrow{a} s' \in T \mid s \in B \wedge s' \in B'\}$.
- The *bottom states* of $B$, i.e., the states without outgoing inert transitions:
  $\mathit{Bottom}(B) = \{s \in B \mid \neg\exists s' \in B. s \xrightarrow{\tau} s'\}$.
- The states in $B$ with a transition in bunch $T$: $B_{\xrightarrow{T}} = \{s \mid s \xrightarrow{a} s' \in T_{B\rightarrow}\}$.
- The outgoing transitions of block $B$: $B_{\rightarrow} = \{s \xrightarrow{a} s' \mid s \in B, a \in \mathit{Act}, s' \in S\}$.
- The incoming transitions of block $B$: $B_{\leftarrow} = \{s \xrightarrow{a} s' \mid s \in S, a \in \mathit{Act}, s' \in B\}$.

The block-bunch-slices and action-block-slices are explicitly maintained as auxiliary data structures in the algorithm in order to meet the required performance bounds. If the partitions $\Pi_s$ or $\Pi_t$ are adapted, all the derived sets above also change accordingly.

A bunch can be *trivial,* which means that it only contains one action-block-slice, or it can contain multiple action-block-slices. In the latter case one action-block-slice is split off to become a bunch by itself. However, this may invalidate Invariant 1. Some states in a block may only have transitions in the new bunch while other states have only transitions in the old bunch. Therefore, blocks have to be split to satisfy Invariant 1. Splitting blocks can cause bunches to become non-trivial because action-block-slices fall apart.

This splitting is repeated until all bunches are trivial, and as already stated above, the obtained partition $\Pi_s$ is the required branching bisimulation. As the transition system is finite this process of repeated splitting terminates.

#### 3.2 Abstract algorithm

We first present an abstract version of the algorithm in Algorithm 1. Its behaviour is as follows. As long as there are non-trivial bunches—i.e, bunches containing multiple action-block-slices—, these bunches need to be split such that they ultimately become trivial. The outer loop (Lines 1.2–1.19) takes a

**Algorithm 1** Abstract algorithm for branching bisimulation partitioning

```
1.1:  Contract τ-SCCs; initialize Π_s and Π_t
1.2:  for all non-trivial bunches T ∈ Π_t do
1.3:      Select an action-block-slice T_{-a->B'} ⊂ T
1.4:      Split T into T_{-a->B'} and T \ T_{-a->B'}
1.5:      for all unstable blocks B ∈ Π_s (i.e., ∅ ≠ T_{B-a->B'} ≠ T_{B->}) do
1.6:          First make T_{B-a->B'} a primary splitter; then make T_{B->} \ T_{B-a->B'} a secondary splitter
1.7:      end for
1.8:      for all splitters T'_{B->} (in order) do
1.9:          Split B into the subblock R that can inertly reach T'_{B->} and the rest U
1.10:         if T'_{B->} was a primary splitter (note: T'_{B->} = T_{B-a->B'}) then
1.11:             Make T_{U->} \ T_{U-a->B'} a non-splitter
1.12:         end if
1.13:         if there are new non-inert transitions R -τ-> U then
1.14:             Split R into the subblock N that can inertly reach R -τ-> U and the rest R'
1.15:             Make all block-bunch-slices T_{N->} of N secondary splitters
1.16:             Create a bunch for the new non-inert transitions (N -τ-> U) ∪ (N -τ-> R')
1.17:         end if
1.18:     end for
1.19: end for
1.20: return Π_s
```

non-trivial bunch $T$ from $\Pi_t$, and from this it moves an action-block-slice $T_{\xrightarrow{a} B'}$ into its own bunch in $\Pi_t$ (Line 1.4). Hence, bunch $T$ is reduced to $T \setminus T_{\xrightarrow{a} B'}$.

The two new bunches $T_{\xrightarrow{a} B'}$ and $T \setminus T_{\xrightarrow{a} B'}$ can cause instability, violating Invariant 1. This means there can be blocks with transitions in one new bunch, but some bottom states only have transitions in the other new bunch. For such blocks, stability needs to be restored by splitting them.

To restore this stability we investigate all block-bunch-slices in one of the new bunches, namely $T_{\xrightarrow{a} B'}$. Blocks that do not have transitions in these block-bunch-slices are stable with respect to both bunches. To keep track of the blocks that still need to be split, we partition the block-bunch-slices $T_{B\rightarrow}$ into *stable* and *unstable* block-bunch-slices. A block-bunch-slice is stable if we have ensured that it is not a splitter for any block. Otherwise it is deemed unstable, and it needs to be checked whether it is stable, or whether the block $B$ must be split. The first inner loop (Lines 1.5–1.7) inserts all unstable block-bunch-slices into the *splitter list.* Block-bunch-slices of the shape $T_{B \xrightarrow{a} B'}$ in the splitter list are labelled *primary,* and other list entries are labelled *secondary.*

In the second loop (Lines 1.8–1.18), one splitter $T'_{B\rightarrow}$ from the splitter list is taken at a time and its source block is split into $R$ (the part that can inertly reach $T'_{B\rightarrow}$) and $U$ (the part that cannot inertly reach $T'_{B\rightarrow}$) to re-establish stability.

If $T'_{B\rightarrow}$ was a primary splitter of the form $T_{B \xrightarrow{a} B'}$, then we know that $U$ must be stable under $T_{U\rightarrow} \setminus T_{U \xrightarrow{a} B'}$, as every bottom state in $B$ has a transition in the former block-bunch-slice $T_{B\rightarrow}$, and as the states in $U$ have no transition in $T_{B \xrightarrow{a} B'}$, every bottom state in $U$ must have a transition in $T_{B\rightarrow} \setminus T_{B \xrightarrow{a} B'}$. Therefore, at Line 1.11, block-bunch-slice $T_{U\rightarrow} \setminus T_{U \xrightarrow{a} B'}$ can be removed from the splitter list. This is the three-way split from [18].

Some inert transitions may have become non-inert, namely the $\tau$-transitions that go from $R$ to $U$. There cannot be $\tau$-transitions from $U$ to $R$. The new non-inert transitions were not yet part of a bunch in $\Pi_t$. So, a new bunch $R \xrightarrow{\tau} U$ is formed for them. All transitions in this new bunch leave $R$ and thus $R$ is the only block that may not be stable under this new bunch. To avoid superfluous work, we split off the unstable part $N$, i.e. the part that can inertly reach a transition in $R \xrightarrow{\tau} U$ and contains all new bottom states, at Line 1.14. The original bottom states of $R$ become the bottom states of $R'$. There can be transitions $N \xrightarrow{\tau} R'$ that also become non-inert, and we add these to the new bunch $R \xrightarrow{\tau} U$. As observed in [10], blocks containing new bottom states can become unstable under any bunch. So, stability of $N$ (but not of $R'$) must be re-established, and all block-bunch-slices leaving $N$ are put on the splitter list at Line 1.15.

#### 3.3 Correctness

The validity of the algorithm follows from a number of major invariants. The main invariant, Invariant 1, is valid at Line 1.2. Additionally, the algorithm satisfies the following three invariants.

**Invariant 2 (Bunches are not unnecessarily split).** *For any pair of non-inert transitions $s \xrightarrow{a} s'$ and $t \xrightarrow{a} t'$, if $s, t \in B$ and $s', t' \in B'$ then $s \xrightarrow{a} s' \in T$ and $t \xrightarrow{a} t' \in T$ for some bunch $T \in \Pi_t$.*

**Invariant 3 (Preservation of branching bisimilarity).** *For all states $s, t \in S$, if $s \leftrightarroweq_b t$, then there is some block $B \in \Pi_s$ such that $s, t \in B$.*

**Invariant 4 (No inert loops).** *There is no inert loop in a block, i.e., for every sequence $s_1 \xrightarrow{\tau} s_2 \xrightarrow{\tau} \cdots \xrightarrow{\tau} s_n$ with $s_i \in B \in \Pi_s$, $n > 1$ it holds that $s_1 \neq s_n$.*

Invariant 2 indicates that two non-inert transitions that (1) start in the same block, (2) have the same label, and (3) end in the same block, always reside in the same bunch. Invariant 3 says that branching bisimilar states never end up in separate blocks. Invariant 4 ensures that all $\tau$-paths in each block are finite. As a consequence every block has at least one bottom state, and from every state a bottom state can be inertly reached.

The invariants given above allow us to prove that the algorithm works correctly. When the algorithm terminates (and this always happens, see Section 3.5), branching bisimilar states are perfectly grouped in blocks.

**Theorem 1.** *From the Invariants 1, 3 and 4, it follows that after the algorithm terminates, $\equiv_{\Pi_s} = \leftrightarroweq_b$.*

Because of the space restrictions here, the proofs are omitted. The interested reader is referred to [14] for the details.

#### 3.4 In-depth description of the algorithm

To show that the algorithm has the desired $O(m \log n)$ time complexity, we now give a more detailed description of the algorithm. The pseudocode of the detailed algorithm is given in Algorithm 2. This algorithm serves two purposes. First of all, it clarifies how the data structures are used, and refines many of the steps in the high-level algorithm. Additionally, time budgets for parts of the algorithm

**Algorithm 2** Detailed algorithm for branching bisimulation partitioning

2.1: Find $\tau$-SCCs and contract each of them to a single state
2.2: $B_{\text{vis}} := \{s \in S \mid s$ can inertly reach some $s' \xrightarrow{a} s''\}$; $B_{\text{invis}} := S \setminus B_{\text{vis}}$
2.3: $\Pi_s := \{B_{\text{vis}}, B_{\text{invis}}\} \setminus \{\emptyset\}$
2.4: $\Pi_t := \{\{s \xrightarrow{a} s' \mid a \in Act \setminus \{\tau\}, s, s' \in S\} \cup B_{\text{vis}} \xrightarrow{\tau} B_{\text{invis}}\}$ } $O(m)$ (lines 2.1–2.4)
2.5: **for all** non-trivial bunches $T \in \Pi_t$ **do** } $\leq m$ iterations
2.6:     Select $a \in Act$ and $B' \in \Pi_s$ with $|T_{\xrightarrow{a} B'}| \leq \frac{1}{2}|T|$
2.7:     $\Pi_t := (\Pi_t \setminus \{T\}) \cup \{T_{\xrightarrow{a} B'}, T \setminus T_{\xrightarrow{a} B'}\}$
2.8:     **for all** unstable blocks $B \in \Pi_s$ with $\emptyset \subset T_{B \xrightarrow{a} B'} \subset T_{B\rightarrow}$ **do**
2.9:         Append $T_{B \xrightarrow{a} B'}$ as primary to the splitter list
2.10:         Append $T_{B\rightarrow} \setminus T_{B \xrightarrow{a} B'}$ as secondary to the splitter list
2.11:         Mark all transitions in $T_{B \xrightarrow{a} B'}$
2.12:         For every state $\in B$ with both marked outgoing transitions and outgoing transitions in $T_{B\rightarrow} \setminus T_{B \xrightarrow{a} B'}$, mark one such transition
2.13:     **end for** } $O(|T_{\xrightarrow{a} B'}|)$ (lines 2.6–2.13)
2.14:     **for all** splitters $T'_{B\rightarrow}$ in the splitter list (in order) **do** } $\leq |T_{\xrightarrow{a} B'}|$ iterations
2.15:         $\langle R, U\rangle := \mathsf{split}(B, T'_{B\rightarrow})$
2.16:         Remove $T'_{B\rightarrow} = T'_{R\rightarrow}$ from the splitter list
2.17:         $\Pi_s := (\Pi_s \setminus \{B\}) \cup (\{R, U\} \setminus \{\emptyset\})$
2.18:         **if** $T'_{B\rightarrow}$ was a primary splitter (note: $T'_{B\rightarrow} = T_{B \xrightarrow{a} B'}$) **then**
2.19:             Remove $T_{U\rightarrow} \setminus T_{U \xrightarrow{a} B'}$ from the splitter list
2.20:         **end if**
2.21:         **if** $R \xrightarrow{\tau} U \neq \emptyset$ **then**
2.22:             Create a new bunch containing exactly $R \xrightarrow{\tau} U$, add $R \xrightarrow{\tau} U = (R \xrightarrow{\tau} U)_{R\rightarrow}$ to the splitter list, and mark all its transitions } $O(|Marked(T'_{B\rightarrow})| + |U_{\rightarrow}| + |U_{\leftarrow}| + |Bottom(N)_{\rightarrow}|)$ or $O(|R_{\rightarrow}| + |R_{\leftarrow}|)$ (lines 2.15–2.22)
2.23:             $\langle N, R'\rangle := \mathsf{split}(R, R \xrightarrow{\tau} U)$
2.24:             Remove $R \xrightarrow{\tau} U = (R \xrightarrow{\tau} U)_{N\rightarrow}$ from the splitter list
2.25:             $\Pi_s := (\Pi_s \setminus \{R\}) \cup (\{N, R'\} \setminus \{\emptyset\})$
2.26:             Add $N \xrightarrow{\tau} R'$ to the bunch containing $R \xrightarrow{\tau} U$ } $O(|R \xrightarrow{\tau} U| + |R'_{\rightarrow}| + |R'_{\leftarrow}| + |Bottom(N)_{\rightarrow}|)$ or $O(|N_{\rightarrow}| + |N_{\leftarrow}|)$ (lines 2.23–2.26)
2.27:             Insert all $T_{N\rightarrow}$ as secondary into the splitter list } $O(|Bottom^*(N)_{\rightarrow}|)$
2.28:             For each bottom state $\in N$, mark one of its outgoing transitions in every $T_{N\rightarrow}$ where it has one } $O(|Bottom(N)_{\rightarrow}|)$
2.29:         **end if**
2.30:     **end for**
2.31: **end for**
2.32: **return** $\Pi_s$

are printed in grey at the right-hand side of the pseudocode. We use these time budgets in Section 3.5 to analyse the overall complexity of the algorithm. We focus on the most important details in the algorithm.

At Lines 2.6–2.7, a *small* action-block-slice $T_{\xrightarrow{a} B'}$ is moved into its own bunch, and $T$ is reduced to $T \setminus T_{\xrightarrow{a} B'}$. All blocks that have transitions in the two new bunches are added to the splitter list in Lines 2.8–2.13. This loop also marks some transitions (in the time complexity annotations we write $Marked(T_{B\rightarrow})$ for the marked transitions of block-bunch-slice $T_{B\rightarrow}$). The function of this marking is similar to that of the counters in [18]: it serves to determine quickly whether a bottom state has a transition in a secondary splitter $T_{B\rightarrow} \setminus T_{B \xrightarrow{a} B'}$ (or slices that are the result of splitting this slice). In general, a bottom state has transitions in some splitter block-bunch-slice if and only if it has marked transitions in this slice. There is one exception: After splitting under a primary splitter $T_{B\rightarrow}$, bottom states in $U$ are not marked. But as they always have a transition in $T_{U\rightarrow} \setminus T_{U \xrightarrow{a} B'}$, $U$ is already stable in this case (see Line 2.19).

The second loop is refined to Lines 2.14–2.30. In every iteration one splitter $T'_{B\rightarrow}$ from the splitter list is considered, and its source block is first split into $R$

and $U$. Formally, the routine $\mathsf{split}(B, T)$ delivers the pair $\langle R, U \rangle$ defined by:

$$R = \{s \in B \mid s \xrightarrow{\tau} s_1 \xrightarrow{\tau} \cdots \xrightarrow{\tau} s_n \xrightarrow{a} s' \text{ where } s_1, \ldots, s_n \in B,\ s_n \xrightarrow{a} s' \in T\},$$
$$U = B \setminus R. \tag{1}$$

We detail its algorithm and discuss its correctness in Section 4.

In Lines 2.21–2.28, the situation is handled when some inert transitions have become non-inert. We mark one of the outgoing transitions of every new bottom state such that we can find the bottom states with a transition in $T_{N\rightarrow}$ in time proportional to the number of such new bottom states.

We illustrate the algorithm in the following example. Note this also illustrates some of the details of the $\mathsf{split}$ subroutine, which is discussed in detail in Section 4.

*Example 1.* Consider the situation in Figure 1a. Observe that block $B$ is stable w.r.t. the bunches $T$ and $T'$. We have split off a small bunch $T_{\xrightarrow{a} B'}$ from $T$, and as a consequence, $B$ needs to be restabilised. The bunches put on the splitter list initially are $T_{\xrightarrow{a} B'}$ and $T \setminus T_{\xrightarrow{a} B'}$. When putting these bunches on the splitter list, all transitions in $T_{B \xrightarrow{a} B'}$ are marked, see the $m$'s in Figure 1b. Also, for states that have transitions both in $T_{\xrightarrow{a} B'}$ and in $T \setminus T_{\xrightarrow{a} B'}$, one transition in the latter bunch is marked, see the $m$'s in Figure 1b.

We now first split $B$ w.r.t. the primary splitter $T_{\xrightarrow{a} B'}$ into $R$, the states that can inertly reach $T_{\xrightarrow{a} B'}$, and $U$, the states that cannot. In Figure 1b, the states known to be destined for $R$ are indicated by ◍, the states known to be destined for $U$ are indicated by ⊜. Initially, all states with a marked outgoing transition are destined for $R$, the remaining bottom state of $B$ is destined for $U$. The $\mathsf{split}$ subroutine proceeds to extend sets $R$ and $U$ in a backwards fashion using two coroutines, marking a state destined for $R$ if one of its successors is already in $R$, and marking a state destined for $U$ if all its successors are in $U$. Here, the state in $U$ does not have any incoming inert transitions, so its coroutine immediately terminates and all other states belong to $R$. Block $B$ is split into subblocks $R$ and $U$, as shown in Figure 1c. Block $U$ is stable w.r.t. both $T_{\xrightarrow{a} B'}$ and $T \setminus T_{\xrightarrow{a} B'}$.

We still need to split $R$ w.r.t. $T \setminus T_{\xrightarrow{a} B'}$, into $R_1$ and $U_1$, say. For this, we use the marked transitions in $T \setminus T_{\xrightarrow{a} B'}$ as a starting point to compute all bottom states that can reach a transition in $T \setminus T_{\xrightarrow{a} B'}$. This guarantees that the time we use is proportional to the size of $T_{\xrightarrow{a} B'}$. Initially, there is one state destined for $R_1$, marked ◍ in Figure 1c, and one state destined for $U_1$, marked ⊜ in the same figure. We now perform the two coroutines in $\mathsf{split}$ simultaneously. Figure 1d shows the situation after both coroutines have considered one transition: The $U_1$-coroutine (which calculates the states that cannot inertly reach $T \setminus T_{\xrightarrow{a} B'}$) has initialised the counter *untested* of one state to 2 on Line 3.9$\ell$ of Algorithm 3 because two of its outgoing inert transitions have not yet been considered. The $R_1$-coroutine (which calculates the states that can inertly reach $T \setminus T_{\xrightarrow{a} B'}$) has checked the unmarked transition in the splitter $T_{R\rightarrow} \setminus T_{R \xrightarrow{a} B'}$. As the latter coroutine has finished visiting unmarked transitions in the splitter, the $U_1$-coroutine no longer needs to run the slow test loop at Lines 3.13$\ell$–3.17$\ell$ of the left column of Algorithm 3. In Figure 1e the situation is shown after two more steps in the coroutines. Each has visited two extra transitions. There two

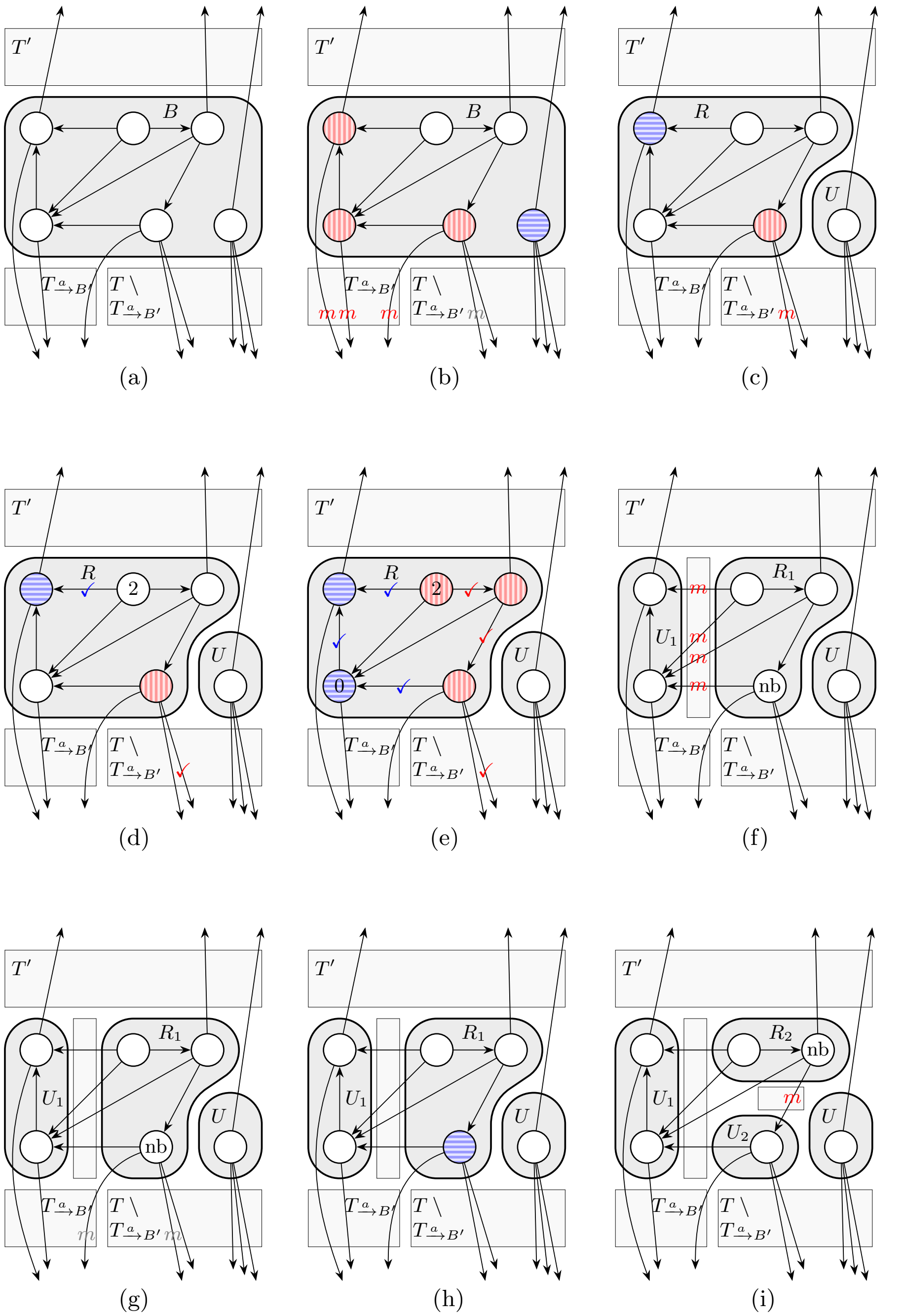

Fig. 1: Illustration of splitting of a small block from $T$ and stabilising block $B$ with respect to the new bunches $T_{\xrightarrow{a} B'}$ and $T \setminus T_{\xrightarrow{a} B'}$, as explained in Example 1.

extra are states destined for $R_1$, marked ◍, and one state is destined for $U_1$ with 0 remaining inert transitions, for which we know immediately that it has no transition in $T \setminus T_{\xrightarrow{a} B'}$, this is marked ⊜. Now, the $R_1$-coroutine is terminated, since it contains more that $\frac{1}{2}|R|$ states, and the remaining incoming transitions of states in $U_1$ are visited. This will not further extend $U_1$. The result of splitting is shown in Figure 1f. Some inert transitions become non-inert, so a new bunch with transitions $R_1 \xrightarrow{\tau} U_1$ is created, and all these transitions are marked $m$.

We next have to split $R_1$ with respect to this new bunch into the set of states $N_1$ that can inertly reach a transition in the new bunch, and the set $R_1'$ that cannot inertly reach this bunch. In this case, all states in $R_1$ have a marked outgoing transition, hence $N_1 = R_1$, and $R_1' = \emptyset$. The coroutine that calculates the set of states that cannot inertly reach a transition in the bunch will immediately terminate because there are no transitions to be considered.

Observe that $R_1$ $(= N_1)$ has a new bottom state, marked 'nb'. This means that stability of $R_1$ with respect to any bunch is not guaranteed any more and needs to be re-established. We therefore consider all bunches in which $R_1$ has an outgoing transition. We add $T_{R_1 \xrightarrow{a} B'}$, $T_{R_1 \rightarrow} \setminus T_{R_1 \xrightarrow{a} B'}$ and $T'_{R_1 \rightarrow}$ to the splitter list as secondary splitters, and mark one outgoing transition from each bottom state in each of these bunches using $m$. This situation is shown in Figure 1g.

In this case, $R_1$ is stable w.r.t. $T_{R_1 \xrightarrow{a} B'}$ and $T_{R_1 \rightarrow} \setminus T_{R_1 \xrightarrow{a} B'}$, i.e., all states in $R_1$ can inertly reach a transition in both bunches. In both cases this is observed immediately after initialisation in split, since the set of states that cannot inertly reach a transition in these bunches is initially empty, and the corresponding coroutine terminates immediately.

Therefore, consider splitting $R_1$ with respect to $T'_{R_1 \rightarrow}$. This leads to $R_2$, the set of states that can inertly reach a transition in $T'$, and $U_2$, the set of states that cannot inertly reach a transition in $T'$. Note there are no marked transitions in $T'_{R_1 \rightarrow}$, so initially all bottom states of $R_1$ are destined for $U_2$ (marked ⊜ in Figure 1h), and there are no states destined for $R_2$. Then we start splitting $R_1$. In the $R_2$-coroutine, we first add the states with an unmarked transition in $T'_{R_1 \rightarrow}$ to $R_2$ at Line 3.4$r$ (i.e., in the right column of Algorithm 3) and then all predecessors of the new bottom state need to be considered. When split terminates, there will be no additional states in $U_2$, and the remaining states end up in $R_2$.

The situation after splitting $R_1$ into $R_2$ and $U_2$ is shown in Figure 1i. One of the inert transitions (marked $m$) becomes non-inert. Furthermore, $R_2$ contains a new bottom state. This is the state with a transition in $T'$. As each block must have a bottom state, a non-bottom state had to become a bottom state.

We need to continue stabilising $R_2$ w.r.t. bunch $R_2 \xrightarrow{\tau} U_2$, which does not lead to a new split, and we need to restabilise $R_2$ w.r.t. all bunches in which it has an outgoing transition. This also does not lead to new splits, so the situation in Figure 1i after removing the markings is the final result of splitting.

### 3.5 Time complexity

Throughout this section, let $n$ be the number of states and $m$ the number of transitions in the LTS. To simplify the complexity notations we assume that

$n \leq m+1$. This is not a significant restriction, since it is satisfied by any LTS in which every non-initial state has an incoming transition. We also write $in(s)$ and $out(s)$ for the sets of incoming and outgoing transitions of state $s$.

We use the principle "Process the smaller half" [13]: when a set is split into two parts, we spend time proportional to the size of the smaller subset. This leads to a logarithmic number of operations assigned to each element. We apply this principle twice, once to new bunches and once to new subblocks. Additionally, we spend some time on new bottom states. This is formulated in the following theorem.

**Theorem 2.** *For the main loop of Algorithm 2 we have:*

1. *A transition is moved to a new small bunch at most* $\lfloor \log_2 n^2 \rfloor + 1$ *times. Whenever this happens, constant time is spent on this transition.*
2. *A state* $s$ *is moved to a new small subblock at most* $\lfloor \log_2 n \rfloor$ *times. Whenever this happens,* $O(|in(s)| + |out(s)| + 1)$ *time is spent on state* $s$*.*
3. *A state* $s$ *becomes a new bottom state at most once. When this happens,* $O(|out(s)| + 1)$ *time is spent on state* $s$*.*

*Summing up these time budgets leads to an overall time complexity of* $O(m \log n)$*.*

These runtimes are annotated as time budgets in the main loop of Algorithm 2. Line 2.7 moves the transitions of $T_{\xrightarrow{a} B'}$ to their new bunch, and Lines 2.6–2.14 take time proportional to the size of this new bunch.

A new subblock is formed at Line 2.17 (and at the same time, some states in subblock $R$ may become new bottom states). Lines 2.15–2.22 take time proportional to its incoming and outgoing transitions. Similarly, a new subblock is formed in Line 2.23, and Lines 2.23–2.26 take time proportional to this subblock's transitions.

Finally, new bottom states found in $R$ (and separated into $N$) allow to spend time proportional to $Bottom(N)_{\rightarrow}$ at Lines 2.15–2.28. At Line 2.27 we need to include not only the current new bottom states but also the future ones because there may be block-bunch-slices that only have transitions from non-bottom states. When $N$ is split under such a block-bunch-slice, at least one of these states will become a bottom state.

Time spent per marked transition fits the time bound because only a small number of transitions is marked: In Lines 2.11 and 2.12, at most two transitions are marked per transition in the small splitter $T_{\xrightarrow{a} B'}$. Line 2.22 marks $R \xrightarrow{\tau} U \subseteq out(R) \cap in(U)$, which is always within the transitions of the smaller subblock. Line 2.28 marks no more transitions than the new bottom states have.

The initialisation in Lines 2.1–2.5 can be performed in $O(m)$ time, where the assumption $n \leq m+1$ is used. Furthermore, we assume that we can access action labels fast enough to bucket sort the transitions in time $O(m)$, which is for instance the case if the action labels are consecutively numbered.

To meet the indicated time budgets, our implementation uses a number of data structures. States are stored in a *refinable partition* [21], grouped per block, in such a way that we can visit bottom states without spending time on non-bottom states. Transitions are stored in four linked refinable partitions, grouped per source state, per target state, per bunch, and per block-bunch-slice, in such a way that we can visit marked transitions without spending time on unmarked transitions of the block. How these data structures are instrumental for the complexity can be found in [14].

### 4 Splitting blocks

The function $\mathsf{split}(B, T)$, presented in Algorithm 3, refines block $B$ into subblocks $R$ and $U$, where $R$ contains those states in $B$ that can inertly reach a transition in $T$, and $U$ contains the states that cannot, as formally specified in Equation (1).

These two sets are computed by two coroutines executing in lockstep: the two coroutines start the same number of loop iterations, so that the overhead is at most proportional to the faster of the two and all work done in both coroutines can be attributed to the smaller of the two subblocks $R$ and $U$.

As a precondition, $\mathsf{split}$ requires that bottom states of $B$ with an outgoing transition in $T_{B\rightarrow}$ have a marked outgoing transition in $T_{B\rightarrow}$. Formally, $\mathit{Bottom}(B)_{\xrightarrow{\mathit{Marked}(T_{B\rightarrow})}} = \mathit{Bottom}(B)_{\xrightarrow{T_{B\rightarrow}}}$. This allows to compute the initial sets: All states in $B_{\xrightarrow{\mathit{Marked}(T)}}$, i.e., sources of marked transitions in $T$, are put in $R$. All bottom states that are not initially in $R$ are put in $U$.

The sets are extended as follows in the coroutines. For $R$, first the states in $B_{\xrightarrow{T \setminus \mathit{Marked}(T)}}$ are added that were not yet in $R$. These are all the sources of unmarked transitions in $T$. Using backward reachability along inert transitions, $R$ is extended until no more states can be added.

**Algorithm 3** Refinement of a block under a splitter

| Line | Coroutine 1 | Coroutine 2 | Complexity |
|---|---|---|---|
| 3.1: | **function** $\mathsf{split}$(block $B$, block-bunch-slice $T$) | | |
| 3.2: | $R := B_{\xrightarrow{\mathit{Marked}(T)}}$; $U := \mathit{Bottom}(B) \setminus R$ | | } $O(\lvert \mathit{Marked}(T) \rvert)$ |
| 3.3: | **begin coroutines** | | |
| 3.4: | Set $\mathit{untested}[t]$ to undefined for all $t \in B$ | $R := R \cup B_{\xrightarrow{T \setminus \mathit{Marked}(T)}}$ | } $O(1)$ or $O(\lvert R_{\rightarrow} \rvert)$ |
| 3.5: | **for all** $s \in U$ **while** $\lvert U \rvert \leq \frac{1}{2} \lvert B \rvert$ **do** | **for all** $s \in R$ **while** | |
| 3.6: | **for all** inert $t \xrightarrow{\tau} s$ **do** | $\lvert R \rvert \leq \frac{1}{2} \lvert B \rvert$ **do** | |
| 3.7: | **if** $t \in R$ **then** Skip $t$, i.e. **goto** 3.6$\ell$ | **for all** inert $t \xrightarrow{\tau} s$ **do** | |
| 3.8: | **if** $\mathit{untested}[t]$ is undefined **then** | | $O(\lvert U_{\leftarrow} \rvert)$ or |
| 3.9: | $\mathit{untested}[t] := \lvert \{ t \xrightarrow{\tau} u \mid u \in B \} \rvert$ | | $O(\lvert R_{\leftarrow} \rvert)$ |
| 3.10: | **end if** | | |
| 3.11: | $\mathit{untested}[t] := \mathit{untested}[t] - 1$ | | |
| 3.12: | **if** $\mathit{untested}[t] > 0$ **then** Skip $t$ | | |
| 3.13: | **if** $B_{\xrightarrow{T}} \not\subseteq R$ **then** | | |
| 3.14: | **for all** non-inert $t \xrightarrow{\alpha} u$ **do** | | $O(\lvert U_{\rightarrow} \rvert +$ |
| 3.15: | **if** $t \xrightarrow{\alpha} u \in T$ **then** Skip $t$ | | $\lvert (\mathit{Bottom}(R) \setminus$ |
| 3.16: | **end for** | | $\mathit{Bottom}(B))_{\rightarrow} \rvert)$ |
| 3.17: | **end if** | | |
| 3.18: | Add $t$ to $U$ | Add $t$ to $R$ | $O(\lvert U_{\leftarrow} \rvert)$ or |
| 3.19: | **end for** | **end for** | $O(\lvert R_{\leftarrow} \rvert)$ |
| 3.20: | **end for** | **end for** | |
| 3.21: | **if** $\lvert U \rvert > \frac{1}{2} \lvert B \rvert$ **then** | **if** $\lvert R \rvert > \frac{1}{2} \lvert B \rvert$ **then** | |
| 3.22: | Abort this coroutine | Abort this coroutine | |
| 3.23: | **end if** | **end if** | $O(1)$ |
| 3.24: | Abort the other coroutine | Abort the other coroutine | |
| 3.25: | **return** $(B \setminus U, U)$ | **return** $(R, B \setminus R)$ | |
| 3.26: | **end coroutines** | | |

To identify the states in $U$, observe that a state is in $U$ if all its inert successors are in $U$ and it does not have a transition in $T_{B\rightarrow}$. To compute $U$, we let a counter $\mathit{untested}[t]$ for every non-bottom state $t$ record the number of outgoing inert transitions to states that are not yet known to be in $U$. If $\mathit{untested}[t] = 0$, this means all inert successors of $t$ are guaranteed to be in $U$, so, provided $t$ does not have a transition in $T_{B\rightarrow}$, one can also add $t$ to $U$. To take care of the possibility that all inert transitions of $t$ have been visited before all sources of unmarked transitions in $T_{B\rightarrow}$ are added to $R$, we check all non-inert transitions of $t$ to determine whether they are not in $T_{B\rightarrow}$ at Lines 3.13ℓ–3.17ℓ.

The coroutine that finishes first, provided that its number of states does not exceed $\frac{1}{2}|B|$, has completely computed the smaller subblock resulting from the refinement, and the other coroutine can be aborted. As soon as the number of states of a coroutine is known to exceed $\frac{1}{2}|B|$, it is aborted, and the other coroutine can continue to identify the smaller subblock. In detail, the runtime complexity of $\langle R, U\rangle := \mathsf{split}(B, T)$ is:

- $O(|R_{\rightarrow}| + |R_{\leftarrow}|)$, if $|R| \leq |U|$, and
- $O(|\mathit{Marked}(T)| + |U_{\rightarrow}| + |U_{\leftarrow}| + |(\mathit{Bottom}(R) \setminus \mathit{Bottom}(B))_{\rightarrow}|)$, if $|U| \leq |R|$.

This complexity is inferred as follows. As we execute the coroutines in lockstep, it suffices to show that the runtime bound for the smaller subblock is satisfied.

In case $|R| \leq |U|$, observe $|\mathit{Marked}(T)| \leq |R_{\rightarrow}|$, so we get $O(|R_{\rightarrow}| + |R_{\leftarrow}|)$ directly from the $R$-coroutine. When $|U| \leq |R|$, we use time in $O(|\mathit{Marked}(T)|)$ for Line 3.2, and we use time in $O(|U_{\leftarrow}|)$ for everything else except Lines 3.13ℓ–3.17ℓ. For these latter lines, we distinguish two cases. If it turns out that $t$ has no transition $t \xrightarrow{\alpha} u \in T$, it is a $U$-state, so we attribute the time to $O(|U_{\rightarrow}|)$. Otherwise, it is an $R$-state that had some inert transitions in $B$, but they all are now in $R \xrightarrow{\tau} U$. So $t$ is a new bottom state, and we attribute the time to the outgoing transitions of new bottom states: $O(|(\mathit{Bottom}(R) \setminus \mathit{Bottom}(B))_{\rightarrow}|)$.

### 5 Experimental evaluation

The new algorithm (JGKW20) has been implemented in the mCRL2 toolset [5] and is available in its 201908.0 release. This toolset also contains implementations of various other algorithms, such as the $O(mn)$ algorithm by Groote and Vaandrager (GV) [10] and the $O(m(\log|\mathit{Act}| + \log n))$ algorithm of [9] (GJKW17). In addition, it offers a sequential implementation of the partition-refinement algorithm using state signatures by Blom and Orzan (BO) [3], which has time complexity $O(n^2 m)$. For each state, BO maintains a signature describing which blocks the state can reach directly via its outgoing transitions.

In this section, we report on the experiments we have conducted to compare GV, BO, GJKW17 and JGKW20 when applied to practical examples. In the experiments the given LTSs are minimised w.r.t. branching bisimilarity. The set of benchmarks consists of all LTSs offered by the VLTS benchmark set[3] with at least 60,000 transitions. Their name ends in "_$\lfloor n/1000\rfloor$_$\lfloor m/1000\rfloor$" and thus

[3] http://cadp.inria.fr/resources/vlts.

Table 1: Running time and memory use results. ▼and ▲: significantly better (worse) than the others.

| model | time | | | | space | | | |
|---|---|---|---|---|---|---|---|---|
| | GV | BO | GJKW17 | JGKW20 | GV | BO | GJKW17 | JGKW20 |
| vasy_40_60 | 24. s | 138. s ▲ | .1 s | .05 s ▼ | 65.5 MB | 60.6 MB | 70 MB | 60 MB |
| vasy_18_73 | .21 s | .37 s ▲ | .11 s | .07 s ▼ | 55.6 MB | 56.7 MB | 50 MB | 50 MB |
| vasy_157_297 | 1.7 s | 2. s | .4 s | .2 s ▼ | 97.3 MB | 94.3 MB | 127.2 MB ▲ | 90 MB |
| vasy_52_318 | .31 s | .9 s ▲ | .2 s | .2 s | 73.4 MB | 90.4 MB | 90.6 MB ▲ | 73.4 MB |
| vasy_83_325 | 2.6 s ▲ | 1.0 s | .9 s | .3 s ▼ | 116.2 MB | .11 GB | 230.5 MB ▲ | .10 GB |
| vasy_116_368 | .9 s | 5. s ▲ | .6 s | .4 s ▼ | 92.8 MB | 110.6 MB | .13 GB ▲ | 90 MB |
| vasy_720_390 | .4 s | .9 s | .6 s | .4 s | 105.2 MB | 103.2 MB | .19 GB ▲ | 95.9 MB ▼ |
| vasy_69_520 | 1.5 s | 4. s ▲ | 2.4 s | .8 s ▼ | .15 GB | .15 GB | 358.1 MB ▲ | 162.0 MB |
| cwi_371_641 | 7.4 s ▲ | 5.9 s | 1. s | .7 s | .17 GB | 229.0 MB ▲ | 185.4 MB | .14 GB ▼ |
| vasy_166_651 | 4.9 s ▲ | 1.9 s | 2. s | .7 s ▼ | 157.5 MB | 141.8 MB | 342.9 MB ▲ | 139.5 MB ▼ |
| cwi_214_684 | 1.4 s | 9. s ▲ | .5 s | .5 s | 140.7 MB | 162.1 MB ▲ | 152.0 MB | .13 GB |
| cwi_142_925 | 1.4 s ▲ | .8 s | 1.0 s | .9 s | 152.5 MB | 117.9 MB ▼ | 156.6 MB ▲ | 152.5 MB |
| vasy_386_1171 | 1.4 s | 2. s ▲ | 1.3 s | .9 s ▼ | 229.2 MB | 210.1 MB ▼ | 273.4 MB ▲ | 228.7 MB |
| vasy_66_1302 | 3. s | 4.7 s | 5. s | 2.2 s ▼ | .23 GB ▼ | 283.1 MB | 618.1 MB ▲ | 268.0 MB |
| vasy_164_1619 | 2.0 s | 5. s ▲ | 3. s | - | .25 GB | 235.4 MB | 262.4 MB | 245.0 MB |
| vasy_65_2621 | 90 s ▲ | 11. s | 20 s | 4.7 s ▼ | .5 GB | 534.7 MB | 1.8 GB ▲ | .5 GB |
| cwi_566_3984 | 8. s | 7. s | 8. s | 6. s | .5 GB | 351.5 MB ▼ | 514.0 MB ▲ | .5 GB |
| vasy_1112_5290 | 10. s | 17. s ▲ | 10 s | 10 s | .8 GB | 720.9 MB | 931.5 MB | .7 GB |
| cwi_2165_8723 | .4 min | 3. min ▲ | - | .3 min | 1.3 GB | 1.8726 GB | 2.1321 GB ▲ | 1.2 GB |
| vasy_6120_11031 | 2. min ▲ | 1.7 min | - | .4 min | 1.8 GB | 1.7379 GB | 3.6596 GB ▲ | 1.5960 GB ▼ |
| vasy_2581_11442 | 10 min ▲ | 3. min | - | - | 1.5999 GB | 1.7434 GB | 4.1299 GB ▲ | 1.4 GB ▼ |
| vasy_574_13561 | 50 s | 56. s | - | - | 1.8835 GB ▲ | 1.5217 GB | 1.5 GB | 1.5 GB |
| vasy_4220_13944 | 30 min ▲ | 5. min | - | .6 min | 2.0965 GB | 2.3188 GB | 5.8661 GB ▲ | 2.0 GB ▼ |
| vasy_4338_15666 | 34. min ▲ | 3. min | 2. min | .8 min ▼ | 2.4043 GB | 2.3559 GB | 5.9888 GB ▲ | 1.8535 GB ▼ |
| cwi_2416_17605 | 30 s | 19. s | 20 s | 20 s | 1.6 GB | 1.5157 GB ▼ | 1.6638 GB | 1.6748 GB ▲ |
| vasy_6020_19353 | 25. s | 40 s ▲ | 6. s | 5. s | 870. MB | 2.3442 GB ▲ | 870. MB | 870. MB |
| vasy_11026_24660 | 50 min ▲ | 20 min | 3. min | 1. min ▼ | 3.6475 GB | 4.0513 GB | 9.6425 GB ▲ | 3.4412 GB ▼ |
| lift6-final | 1.0 min | 3. min ▲ | - | .9 min | 3.3846 GB | 8.1984 GB ▲ | 6.2971 GB | 3.2125 GB ▼ |
| vasy_12323_27667 | 40 min ▲ | 10 min | - | 1. min | 4.0091 GB | 4.5371 GB | 10.6743 GB ▲ | 3.7298 GB ▼ |
| vasy_8082_42933 | 2. min | 5. min ▲ | - | 2. min | 6.1231 GB | 5.4358 GB ▼ | 6.6896 GB ▲ | 5.4600 GB |
| cwi_7838_59101 | 5. min | 100 min ▲ | 6. min | 3. min | 6.5283 GB ▼ | 8.3266 GB | 13.7899 GB ▲ | 6.7646 GB |
| dining_14 | 17. min | 20 min | 20 min | 10 min | 20.4826 GB ▼ | 21.7156 GB | 23.7810 GB ▲ | 20.9756 GB |
| cwi_33949_165318 | 11. min | 80 min ▲ | 20 min | 8. min | 22.7204 GB | 33.0351 GB | 37.8606 GB ▲ | 21.0611 GB ▼ |
| 1394-fin3 | 25. h ▲ | 3. h | .5 h | .3 h ▼ | 37.4893 GB | 71.8698 GB ▲ | 53.2166 GB | 31.5132 GB ▼ |
| **Total** | **28. h** ▲ | **8. h** | **1.4 h** | **.8 h** ▼ | **121.8 GB** | **176.33 GB** | **194.2 GB** ▲ | **112.0 GB** ▼ |

describes their size. Additionally, we consider three cases that have been derived from models distributed with the mCRL2 toolset:

1. **lift6-final**: this model is based on an elevator model, extended to six elevators ($n$ = 6,047,527, $m$ = 26,539,368);
2. **dining_14**: this is the dining philosophers model with 14 philosophers ($n$ = 18,378,370, $m$ = 164,329,284);
3. **1394-fin3**: this is an altered version of the 1394-fin model, extended to three processes and two data elements ($n$ = 126,713,623, $m$ = 276,426,688).

The software and benchmarks used for the experiments are available online [15]. All experiments have been conducted on individual nodes of the DAS-5 cluster [1]. Each of these nodes was running CentOS Linux 7.4, had an Intel Xeon E5-2698-v3 2.3GHz CPU, and was equipped with 256 GB RAM. Development version 201808.0.c59cfd413f of mCRL2 was used for the experiments.[4]

Table 1 presents the obtained results. Benchmarks are ordered by their number of transitions. On each benchmark, we have applied each algorithm ten times, and report the mean runtime and memory use of these ten runs, rounded to significant digits (estimated using [4] for the standard deviation). A trailing decimal dot indicates that the unit digit is significant. If this dot is missing, there is one insignificant zero. For all presented data the estimated standard deviation is less than 20% of the mean. Otherwise we print '-' in Table 1.

The ▼-symbol after a table entry indicates that the measurement is significantly better than the corresponding measurements for the other three algorithms, and the ▲-symbol indicates that it is significantly worse. Here, the results are considered significant if, given a hundred tables such as Table 1, one table of running time (resp. memory) is expected to contain spuriously significant results.

Concerning the runtimes, clearly, GV and BO perform significantly worse than the other two algorithms, and JGKW20 in many cases performs significantly better than the others. In particular, JGKW20 is about 40% faster than GJKW17, the fastest older algorithm. Concerning memory use, in the majority of cases GJKW17 uses more memory than the others, while sometimes BO is the most memory-hungry. JGKW20 is much more competitive, in many cases even outperforming every other algorithm.

The results show that when applied to practical cases, JGKW20 is generally the fastest algorithm, and even when other algorithms have similar runtimes, it uses almost always the least memory. This combination makes JGKW20 currently the best option for branching bisimulation minimisation of LTSs.

**Data Availability Statement and Acknowledgement.** The datasets generated and analysed during the current study are available in the figshare repository: https://doi.org/10.6084/m9.figshare.11876688.v1. This work is partly done during a visit of the first author at Eindhoven University of Technology, and a visit of the second author at the Institute of Software, Chinese Academy of Sciences. The first author is supported by the National Natural Science Foundation of China, Grant No. 61761136011.

[4] https://github.com/mCRL2org/mCRL2/commit/c59cfd413f
